# Supplementary material for: Transcriptomic Insights into Mechanisms of Early Seed Maturation in the Garden Pea (Pisum sativum L.)
Source: Cells. 2020 Mar 23;9(3):779. doi: 10.3390/cells9030779 (PMC7140803; doi:10.3390/cells9030779)

Zhongwan−6(25)

Global Summary

%DE = 0.83

genes with fdr < 0.2 = 1718 (583 + / 1135 −)

genes with fdr < 0.1 = 1522 (477 + / 1045 −)

genes with fdr < 0.05 = 1353 (396 + / 957 −)

genes with fdr < 0.01 = 1045 (233 + / 812 −)

genes in genesets = 0

<FC> = 0

<t−score> = −69513.47

<p−value> = 0

<fdr> = 0.17

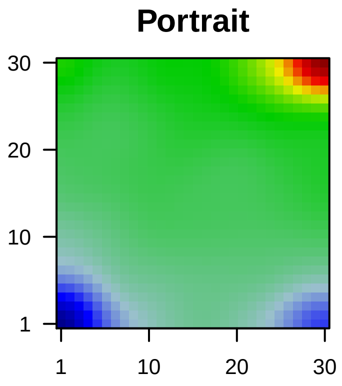

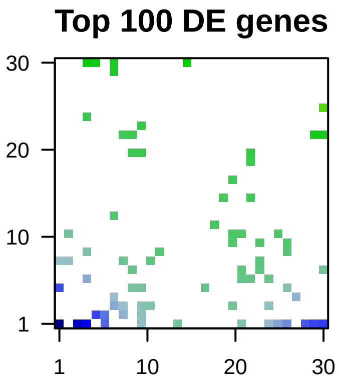

Global Genelist

Rank	ID	log(FC)	p−value	fdr	Metagene	Description
1	comp32197_c	−0.19	0e+00	6e−14	11 x 3	
2	comp32560_c	−0.11	0e+00	6e−14	4 x 6	
3	comp41855_c	−0.19	0e+00	6e−14	1 x 5	
4	comp46799_c	−0.01	0e+00	6e−14	9 x 20	
5	comp31500_c	−0.07	4e−16	8e−14	7 x 3	
6	comp43362_c	−0.07	4e−16	8e−14	7 x 4	
7	comp45569_c	−0.11	4e−16	8e−14	7 x 29	
8	comp22509_c	−0.06	7e−16	1e−13	8 x 2	
9	comp20228_c	−0.03	1e−15	1e−13	10 x 5	
10	comp38522_c	−0.04	1e−15	1e−13	22 x 15	
11	comp41910_c	−0.04	2e−15	1e−13	9 x 5	
12	TRINITY_DN5	−1.68	2e−15	2e−13	30 x 1	
13	TRINITY_DN1	−1.41	3e−15	2e−12	1 x 1	
14	TRINITY_DN2	−1.15	3e−15	2e−12	1 x 1	
15	comp24517_c	−0.02	2e−14	2e−12	11 x 8	
16	TRINITY_DN3	−0.48	2e−14	2e−12	1 x 1	
17	TRINITY_DN3	−0.3	3e−14	2e−12	1 x 5	
18	TRINITY_DN1	−0.13	3e−14	2e−12	6 x 1	
19	TRINITY_DN6	−0.43	3e−14	1e−11	1 x 1	
20	TRINITY_DN7	−0.48	7e−14	2e−11	30 x 1	

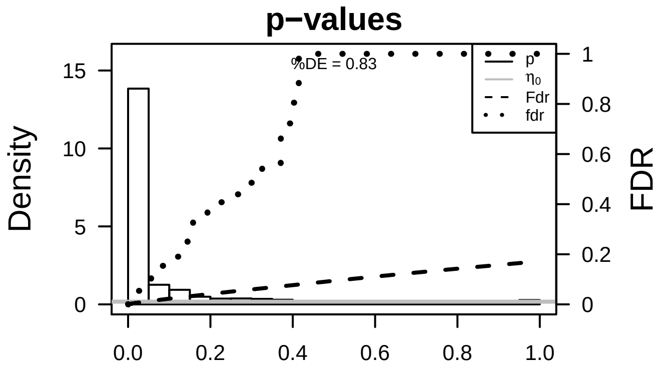

Supplement: Supplementary file 1 [file cells-09-00779-s001.zip › Supplementary materials/FileS4.zip!/FileS3/TFs - Results/Summary Sheets - Groups/Reports/Zhongwan.6.25..pdf]
